# MT5-MMP is a new pro-amyloidogenic proteinase that promotes amyloid pathology and cognitive decline in a transgenic mouse model of Alzheimer’s disease

**DOI:** 10.1007/s00018-015-1992-1

**Published:** 2015-07-23

**Authors:** Kévin Baranger, Yannick Marchalant, Amandine E. Bonnet, Nadine Crouzin, Alex Carrete, Jean-Michel Paumier, Nathalie A. Py, Anne Bernard, Charlotte Bauer, Eliane Charrat, Katrin Moschke, Mothoharu Seiki, Michel Vignes, Stefan F. Lichtenthaler, Frédéric Checler, Michel Khrestchatisky, Santiago Rivera

**Affiliations:** 1 Aix-Marseille Université, CNRS, NICN UMR 7259, 13344 Marseille, France; 2 Labex DistAlz, IPMC UMR 7275 CNRS-UNS, 06560 Valbonne, France; 3 German Center for Neurodegenerative Diseases (DZNE) and Neuroproteomics, Munich, Germany; 4 Klinikum rechts der Isar, and Institute for Advanced Study, Technische Universität München (TUM), 81675 Munich, Germany; 5 Division of Cancer Cell Research, Institute of Medical Science, University of Tokyo, Shirokanedai, Minato-ku, Tokyo 108-8639 Japan; 6 UMR5247 IBMM CNRS University of Montpellier 1 and University of Montpellier 2, 34095 Montepellier, France; 7 Munich Cluster for Systems Neurology (SyNergy), 80336 Munich, Germany; 8 Present Address: Psychology Department, Central Michigan University, Mount Pleasant, MI 48859 USA

**Keywords:** MMP-24, Synaptotoxicity, Neuroprotection, Knockout mouse, Neurodegenerative disease

## Abstract

Membrane-type 5-matrix metalloproteinase (MT5-MMP) is a proteinase mainly expressed in the nervous system with emerging roles in brain pathophysiology. The implication of MT5-MMP in Alzheimer’s disease (AD), notably its interplay with the amyloidogenic process, remains elusive. Accordingly, we crossed the genetically engineered 5xFAD mouse model of AD with MT5-MMP-deficient mice and examined the impact of MT5-MMP deficiency in bigenic 5xFAD/MT5-MMP^−/−^ mice. At early stages (4 months) of the pathology, the levels of amyloid beta peptide (Aβ) and its amyloid precursor protein (APP) C-terminal fragment C99 were largely reduced in the cortex and hippocampus of 5xFAD/MT5-MMP^−/−^, compared to 5xFAD mice. Reduced amyloidosis in bigenic mice was concomitant with decreased glial reactivity and interleukin-1β (IL-1β) levels, and the preservation of long-term potentiation (LTP) and spatial learning, without changes in the activity of α-, β- and γ-secretases. The positive impact of MT5-MMP deficiency was still noticeable at 16 months of age, as illustrated by reduced amyloid burden and gliosis, and a better preservation of the cortical neuronal network and synaptophysin levels in bigenic mice. MT5-MMP expressed in HEKswe cells colocalized and co-immunoprecipitated with APP and significantly increased the levels of Aβ and C99. MT5-MMP also promoted the release of a soluble APP fragment of 95 kDa (sAPP95) in HEKswe cells. sAPP95 levels were significantly reduced in brain homogenates of 5xFAD/MT5-MMP^−/−^ mice, supporting altogether the idea that MT5-MMP influences APP processing. MT5-MMP emerges as a new pro-amyloidogenic regulator of APP metabolism, whose deficiency alleviates amyloid pathology, neuroinflammation and cognitive decline.

**Electronic supplementary material:**

The online version of this article (doi:10.1007/s00018-015-1992-1) contains supplementary material, which is available to authorized users.

## Introduction

Alzheimer’s disease (AD) is the most common neurodegenerative disorder. No cure or prevention exists to date. Proteolytic control of amyloid precursor protein (APP) is central to AD pathogenesis. The sequential cleavage of APP by β-secretase (β-site APP-cleaving enzyme, BACE) and γ-secretase complex releases the amyloid beta peptide (Aβ), whose accumulation is paramount in AD [1]. The alternative α-secretase cleavage of APP by a disintegrin and metalloproteinase-10 (ADAM-10) releases an N-terminal soluble APP fragment (sAPPα), thereby preventing Aβ formation [2]. β- and γ-secretase inhibitors prevent Aβ accumulation and AD pathogenesis in animal models, but they also interfere with the physiological processing of other substrates [3, 4]. It is therefore crucial to identify new targets to restrain APP metabolism and Aβ production while preventing side effects.

Matrix metalloproteinases (MMPs) have pivotal roles in neuroinflammation, neuroplasticity and neural cell death, but their implication in AD remains elusive [5, 6]. MMP-2 and MMP-9 have putative Aβ-degrading activities [1, 5], although MMP-9 overexpression also upregulates sAPPα levels and decreases Aβ oligomers in 5xFAD mice [7, 8], suggesting that overall MMPs could convey beneficial effects in AD. This general assumption is challenged by recent data indicating that membrane-type 1-MMP (MT1-MMP) promotes the accumulation of Aβ and its neurotoxic precursor, the APP C-terminal fragment of 99 amino acids (C99) [9]. Interestingly, MT1-MMP and other members of the MT-MMP subfamily such as MT3- and MT5-MMP can process APP in heterologous cell systems and release soluble APP N-terminal fragments, which are different from those generated by α- and β-secretases [10, 11]. MT5-MMP (also known as MMP-24) stands out as the only MMP essentially expressed in the nervous system [12, 13], whose functional diversity includes the modulation of post-lesion neuronal plasticity [14], neural stem cell proliferation [15], or neuro-immune interactions between neurons and mast cells [16]. MT5-MMP has been detected in brain amyloid plaques of Alzheimer patients [17] and suggested to cooperate with γ-secretase to synergistically hamper synaptic function in neuronal cultures [18]. Collectively, the alleged interactions of MT5-MMP with key components of the amyloidogenic pathway provide strong rationale to question the possible implication of this MMP in AD pathogenesis. Accordingly, we crossed the APP/PS1 transgenic 5xFAD mouse model of AD (Tg thereafter) [19] with mice deficient in MT5-MMP (MT5^−/−^) [14] to generate a new bigenic TgMT5^−/−^ strain and evaluate the impact of MT5-MMP deficiency in this AD mouse model. Compared to Tg mice, TgMT5^−/−^ mice exhibited at early stages of the pathology strong and durable reductions of Aβ load and neuroinflammation along with improved hippocampal LTP and spatial memory, while the activity of the main secretases remained unchanged. Combined in cellulo and in vivo work supported the ability of MT5-MMP to interact with APP and to promote C99 and Aβ accumulation as well as the alternative processing of APP resulting in sAPP95 release. MT5-MMP emerges as proteinase with pro-amyloidogenic features that could be targeted to reduce amyloidosis/neuroinflammation and their associated functional deficits.

## Materials and methods

### Mice

5xFAD transgenic mice (Tg) were purchased from the Jackson Laboratory (Bar Harbor, ME, USA) [19]. They carry three mutations in the human *APP* gene and two mutations in the human *presenilin 1* (*PSEN1*) gene; in the *APP* gene: Swedish (K670N/M671L), Florida (I716V) and London (V717I); in the *PSEN1* gene: M146L and L286V. All transgenes are under transcriptional control of neuron-specific mouse Thy1 promoter. Tg mice (B6/SJL genetic background) were first derived in a C57BL6 genetic background. F1 male mice resulting from crossing Tg B6/SJL and C57Bl6 mice were then crossed with C57Bl6 females and the male offspring backcrossed eight times with C57Bl6 females. The resulting C57Bl6 Tg mice were then crossed with C57BL6 homozygous transgenic MT5-MMP^−/−^ mice [14] to obtain 5xFAD/MT5-MMP^+/−^ and then with MT5-MMP^−/−^ mice to generate 5xFAD/MT5-MMP^−/−^ bigenic mice (TgMT5^−/−^) in a C57BL6 background. Genotyping was performed by PCR analysis of tail DNA [9]. All experimental procedures were conducted only on male mice in accordance with the National and European regulations (EU directive No. 2010/63) and in agreement with the authorization for animal experimentation attributed to the laboratory by the Prefecture des Bouches du Rhone (permit number: D 13 055 08). All efforts were made to minimize animal suffering and to reduce the number of mice used.

### Immunohistochemistry, image analysis and quantifications

Male mice were deeply anesthetized with sodium pentobarbital and transcardially perfused with cold saline (NaCl 0.9 %), followed by 4 % paraformaldehyde in 0.1 M phosphate buffer, pH 7.4 (PFA). The brains were then post-fixed 24 h in the same fixative and stored at 4 °C in phosphate buffer saline (PBS), pH 7.4. Free-floating coronal sections (30 μm thick) were obtained using a vibratome (HM 650V, Thermo Scientific, MA, USA) and stored at −20 °C in a cryoprotectant solution (40 % phosphate buffer 0.5 M, pH 7.4, 30 % ethylene glycol and 30 % glycerol). Sections were pre-incubated in a blocking PBS solution containing 3 % BSA and 0.1 % Triton X-100, followed by overnight incubation at 4 °C with polyclonal anti-GFAP (1/300, Dako France, Trappes, France), monoclonal anti-GFAP (Millipore, Molsheim, France), polyclonal anti-Iba1 (1/200, Wako, Sobodia, Montbonnot-Saint-Martin, France), monoclonal anti-Aβ 6E10 (1/300, Covance, Eurogentec, Angers, France), polyclonal anti-IL-1β (1/200, Peprotech, Neuilly-sur-Seine, France), monoclonal anti-MAP-2 (1/500, Abcam, Paris, France) and monoclonal anti-synaptophysin (1/500, Millipore), and then the corresponding secondary Alexa Fluor^®^ 488 and 568-coupled antibodies (1/800, Life Technologies, Saint Aubin, France). Nuclei were stained with 0.5 µg/ml DNA intercalant Hoechst #33258 (Life Technologies). Sections were mounted using Prolong Gold Antifading reagent (Life Technologies). Omission of primary antibodies was used as negative controls of immunostaining. Tissues were observed under LSM 700 confocal or Axiovert inverted microscopes (Zeiss, Jena, Germany). Images were analyzed using the Axiovision (Zeiss), Photoshop and ImageJ (NIH) softwares.

The number of plaques was blindly scored in two brain sections per animal by three investigators at bregma −1.9 and −2.9 and normalized by the surface of the brain region analyzed. Densitometric analysis of immunostained sections was performed using ImageJ software and binarization methods, applying the same threshold of immunofluorescence across experimental groups. Brain areas of interest were outlined and the percent of immunoreactive pixels scored. The number of glial cells in the vicinity of plaques was determined over a 0.01 mm^2^ area, with the plaque placed at the center of the square.

### Thioflavin T staining

After 1 h in a blocking PBS solution containing 3 % BSA and 0.1 % Triton X-100, free-floating sections were rinsed with PBS and incubated with Hoechst #33258 for 20 min, washed in PBS and then incubated for 15 min under agitation with 10 µg/ml thioflavin T (ThT, Sigma-Aldrich, Saint-Quentin Fallavier, France). Sections were incubated in 70° EtOH for 5 min to neutralize ThT and then washed to remove EtOH. Sections were protected from light and kept at room temperature during the entire procedure and then mounted using Prolong Gold Antifade reagent.

### Western blots

Male mice were deeply anesthetized with sodium pentobarbital and transcardially perfused with cold saline (NaCl 0.9 %). The neocortex and hippocampus were microdissected and snap frozen for biochemical assays. Samples were homogenized in 25 % w/v of 50 mM Tris–HCl buffer, pH 7.5 containing 150 mM NaCl, 2 mM EDTA, 1 % Triton X-100, 0.05 % SDS and a proteinase inhibitor cocktail (Millipore) and centrifuged at 10,000×*g* for 10 min at 4 °C. This “soluble fraction” contained cytosolic proteins and proteins easy to solubilize. Cell pellets were resuspended in 25 % w/v of 50 mM Tris–HCl buffer, pH 7.5 containing 2 % SDS, then sonicated and centrifuged at 10,000×*g* for 10 min at 4 °C to obtain an “insoluble fraction” containing more insoluble proteins, including an important part of membrane proteins [20]. Protein concentrations were determined using a Bio-Rad *DC*™ protein assay kit (Bio-Rad, Marnes-La-Coquette, France) and 50 μg of protein was run on 10–15 % SDS-PAGE gels and transferred onto nitrocellulose membranes (Amersham Bioscience, Velizy-Villacoublay, France) [9]. After blocking, membranes were probed with the antibodies, monoclonal anti-APP N-terminal (22C11, 1/500, Millipore), monoclonal anti-Aβ (6E10, 1/200), polyclonal anti-APP C-terminal (APP-CTF, 1/2000, Sigma-Aldrich), polyclonal anti-MMP-2 (1/500, Millipore), polyclonal anti-MMP-9 (1/500, Millipore), monoclonal anti-synaptophysin (1/1000), monoclonal anti-N-cadherin C-terminal (1/1000, BD Biosciences, Le Pont de Claix, France), monoclonal anti-GAPDH (1/500, Millipore) and monoclonal anti-actin (1/5000, Sigma-Aldrich), and then incubated with horseradish peroxidase-conjugated secondary IgG antibodies (Jackson Immunoresearch, West Grove, PA, USA). MT5-MMP deficiency was confirmed by western blot in the soluble fraction of the hippocampus and cerebellum from Tg and TgMT5^−/−^ mice using a specific rabbit polyclonal MT5-MMP antibody (1/500) developed in our laboratory (Online Resource 1). Immunoblot signals were visualized using the ECL chemiluminescence kit (GE Healthcare, Dutscher, Brumath, France) and quantified using the ImageJ software.

The secreted forms of human APP, hsAPPα and hsAPPβ were analyzed in soluble diethylamine (DEA)-extracted fractions generated according to previously reported protocols [21, 22], using monoclonal anti-hAPPs-α, clone 14D6 and anti-hAPPs-β swe, clone 192swe (kindly provided by Dale Schenk), respectively. Polyclonal anti-calnexin (Enzo Life Sciences Inc., Farmingdale, NY, USA) was used for sample normalization. Blots were developed using appropriate horseradish peroxidase-conjugated secondary IgG antibodies (anti-mouse/anti-rabbit, Promega Corp., Madison, WI, USA; anti-rat, Santa Cruz Biotechnology Inc., Dallas, TX, USA) and the ECL chemiluminescence system (GE Healthcare). Quantification of sAPPα and sAPPβ bands was carried out using Fuji Las-4000 software (Fuji Film inc., Minato, Tokyo, Japan).

Levels of PS1, Aph1, Pen2 and nicastrin were analyzed in solubilized cell membranes as reported previously [23]. Briefly, intact cell pellets were resuspended in 10 mM Tris–HCl buffer, pH 7.5 with proteinases inhibitors (Sigma-Aldrich), subjected to repeated passages through a 25 G needle and first centrifuged at 800×*g* for 10 min at 4 °C. The resulting supernatants were subjected to an additional 20,000×*g* centrifugation for 1 h at 4 °C. Pellets containing the membranes were then resuspended in solubilization buffer, 150 mM sodium citrate, pH 6.4 with 3-[(3-cholamidopropyl) dimethylammonio]-2-hydroxy-1-propanesulfonate [CHAPSO, 1 % (v/v)] and proteinase inhibitors (Millipore). The solubilized membranes were diluted in solubilization buffer (1 mg/ml). The denatured samples were subjected to western blot using a polyclonal anti-PS1 N-terminal (1/1000, provided by Paul Fraser), polyclonal anti-Aph1 (1/1000), polyclonal anti-Pen2 (1/1000, Covance) and polyclonal anti-nicastrin (1/1000, Sigma-Aldrich) and normalized with tubulin using monoclonal anti-tubulin (1/5000, Sigma-Aldrich).

### ELISA assay for Aβ and IL-1β quantifications

For detection of soluble Aβ38, Aβ40 and Aβ42 species in the DEA fractions, we used the MSD Aβ Triplex sandwich immunoassay (MesoScale Discovery, Rockville, MD, USA) [21]. MSD C-terminal-specific antibodies were pre-spotted into each well. For detection, a ruthenylated 4G8 antibody was used. The concentrations of Aβ peptides were normalized to protein content and calculated using the MSD Discovery Workbench software.

For detection of IL-1β in the soluble fraction, we used the murine IL-1β ELISA Development Kit (PeproTech, Neuilly-sur-Seine, France), loading 5 μg of protein per well, according to the manufacturer’s recommendations.

### Plasmid construction

The MT5-MMP cDNA was amplified by PCR from C57Bl6 mouse cerebellum and cloned using protocols previously described for other MMP cDNAs [24–26]. The following primers were used: *MT5For* ATA TAT GAA TTC GGA TGC CGA GGA GCC GGG GAG GCC GCG CTG and *MT5Rev* ATA TAT GTC GAC AGT ACC CAC TCC TGG ACC GGC CGC TTA TAG TAG and cloned into pEGFP-N1 (Clontech, Saint-Germain-en-Laye, France). The intracellular domain (ic), corresponding to the last 22 amino acids, was subcloned after GFP encoding sequence using the following primers: *icFor* TAA TTA CTG TAC AAG AAG AAC AAG GCG GGT CCT CAG C and *icRev* TAT ATA GCG GCC GCT CAT ACC CAC TCC TGG ACC GGC. This construction is referred to as MT5. Inactivation of MT5-MMP (E to Q mutation, referred to as MT5Δ) was performed using the Quick Change Lighting MultiSite-directed Mutagenesis kit (Agilent Technologies, Les Ulis, France) with the primer CCA TGA TAG CAC TGG GGT CAT TAG AGT GCT CCA AGC CCA GTG CAT GGC CCA GTT GAT GCA CGG CCA CCA GGA A, according to the manufacturer’s recommendations. GFP, MT5 and MT5Δ plasmids were amplified in *Escherichia coli* DH5α (Life Technologies) and purified using the NucleoBond Xtra Midi Plus EF (Macherey-Nagel, Hoerd, France) according to the manufacturer’s recommendations.

The TfR cDNA was amplified by PCR from human brain total RNA (Clontech), and cloned into pEGFP-C1 (Clontech). The following primers were used: *TfRFor* ATA TAT GAA TTC TAT GAT GGA TCA AGC TAG ATC AGC ATT CTC T and *TfRev* TTA ATT GTC GAC CTA AAA CTC ATT GTC AAG GTC CCA AAC GTC AC.

### Cell culture and immunoprecipitation assay

Human embryonic kidney cells expressing *APP* carrying the Swedish mutation (HEKswe) were used to assess the interactions of MT5-MMP and its potential amyloidogenic properties in vitro. Like in the 5xFAD mice, the Swedish mutation increases the affinity of BACE1 for APP, which results in higher Aβ production [27]. Cells were transiently transfected using the JetPei^®^ transfection reagent according to the manufacturer’s instructions (Ozyme, Saint Quentin, France). Briefly, HEKswe cells were plated to 10^6^ cells/ml for 24 h in six-well plates in DMEM Glutamax, 10 % FBS (fetal bovine serum) and 1 % penicillin/streptomycin (Life Technologies) and then transfected with 1 μg of GFP, MT5 or TfR plasmids for 16 h. The medium was replaced by OptiMEM containing ITS (Life Technologies) for 48 h. Cell lysates were prepared by sonication in RIPA buffer (Sigma-Aldrich) containing proteinase inhibitors (Millipore). Two hundred and fifty micrograms of proteins in a total volume of 500 μL of RIPA buffer was incubated overnight at 4 °C under agitation with 5 µg of non-specific mouse IgG antibodies (Jackson Immunoresearch) or mouse anti-GFP (Roche Diagnostics, Meylan, France) or anti-APP-CTF (Sigma-Aldrich) antibodies, and then pulled down for 2 h using protein G-coupled Dynabeads (Life Technologies). Samples were boiled and subjected to western blotting using 22C11 (1/1000) and APP-CTF (1/2000) antibodies, polyclonal anti-MT5-MMP (1/1000; developed in our laboratory) and polyclonal anti-transferrin receptor (1/250, Santa Cruz Biotechnology, Inc., Heidelberg, Germany). The latter was used as a putative negative control to demonstrate that overexpressed MT5-MMP/GFP does not inevitably interact in a non-specific manner with any endogenous or overexpressed transmembrane glycoprotein, such as the transferrin receptor (TfR; ~90 kDa).

### Immunocytochemistry and co-localization in HEKswe cells

Double immunocytochemistry was performed on HEKswe cells to determine the colocalization of MT5-MMP/GFP and APP 48 h after transfection, as indicated above. Cells were fixed for 20 min with Antigenfix solution (Diapath, MM France, Francheville, France) at room temperature. After washing in PBS, cells were blocked for 1 h at room temperature using a PBS solution containing 0.1 % Triton X-100 and 3 % BSA. Cells were then incubated overnight at 4 °C with polyclonal anti-GFP (1/500) and 22C11 (1/500) antibodies, followed by incubation with appropriate Alexa Fluor^®^-coupled secondary antibodies for 1 h at room temperature (Life Technologies). Nuclei were stained with Hoechst #33258 (0.5 µg/ml, Life Technologies). Omission of the primary antibody was used as control and no immunostaining was observed. Coverslips were mounted using Prolong Gold Antifading reagent on Superfrost glass slides (Life Technologies). Images were taken and processed using a confocal microscope (LSM 700) and the Zen software (Zeiss). Co-localization analyses were performed using the Jacop plugin of ImageJ [28].

### Aβ production assay

Aβ monomers were identified by immmunoprecipitation and western blot, as previously described [29]. HEKswe cells were plated to 10^6^ cells/ml for 24 h in six-well plates in DMEM Glutamax, 10 % FBS and 1 % penicillin/streptomycin (Life Technologies). The medium was discarded and replaced with OptiMEM containing 1 % FBS; then the cells were transfected with 1 μg of GFP and MT5-MMP/GFP by MT5 plasmids for 16 h [23]. A hundred microliters of 100 mM Tris–HCl buffer, pH 8, containing 1.5 M NaCl and 50 mM EDTA was added to supernatants and then incubated overnight with polyclonal FCA40 antibody and protein A agarose beads (Life Technologies) for immunoprecipitation. Beads were washed twice, boiled and subjected to western blot. Aβ peptide was detected using the 6E10 antibody (1/200). Immunoblot signals were visualized using the ECL chemiluminescence kit (GE Healthcare, Dutscher, Brumath, France) and optical density was quantified using the ImageJ software.

### Electrophysiological recordings

Experiments were performed on hippocampal slices (300 µm) obtained from 4-month-old male mice. After decapitation, the brains were quickly dissected and placed in ice-cold buffer containing: 124 mM NaCl, 3.5 mM KCl, 25 mM NaHCO_3_, 1.25 mM NaH_2_PO_4_, 1 mM CaCl_2_, 2 mM MgSO_4_, 10 mM Glucose and 10 mM Hepes (bubbled with O_2_/CO_2_: 95/5 %). Slices were then prepared with a vibratome (VT1000S Leica, Germany) and maintained at room temperature for at least 1 h in the same buffer supplemented with 1 mM CaCl_2_, which was further used for recordings.

For electrophysiological recordings, slices were transferred to a multielectrode array (MEA60; Multi Channel Systems, Germany) comprising 60 extracellular electrodes [30, 31]. The interelectrode distance was 200 μm. Each individual electrode from the array could be used either as a recording or a stimulating electrode. A nylon mesh was positioned above the slice to obtain a satisfactory electrical contact with the electrode array. Slices were continually superfused with the extracellular medium described above (flow rate 2 ml/min) and kept at 32 °C. Stimulation was achieved with an external stimulator (STG 1004; Multi Channel Systems) by applying biphasic current pulses to one electrode of the array in the Schaffer collateral pathway of the hippocampus. Stimulation intensity (60–200 μA) and duration (70–150 μs) were adapted to avoid multiphasic responses due to an excessive stimulation [32]. Selected electrodes of the array were then used to simultaneously record field excitatory postsynaptic potentials (fEPSPs) in the *stratum radiatum* of the CA1 hippocampal area. Test frequency to evoke fEPSPs was 0.066 Hz. Slices that displayed epileptic-like activity were discarded. Data storage and analysis were performed with MC Rack software (Multi Channel Systems). Changes in synaptic transmission were assessed by measuring peak amplitudes of fEPSPs, which were modified in a way similar to fEPSP slopes, as previously reported by others using MEAs [33]. Applying a single train of stimulation at 100 Hz for 1 s triggered LTP.

### Behavioral tests

Hippocampal-dependent spatial memory was assessed in a six-arm radial water maze (6-ARWM), using a protocol slightly modified with respect to the original method [34]. The maze was inserted in a 120 cm diameter polycarbonate pool containing water at 20 °C, and distal cues of different shapes and sizes were placed on the walls of the testing room. Briefly, 10–12 mice/group were isolated 1 week before testing. Tests were performed during two consecutive days (15 trials/day). Each full entry in the non-goal arm (or 15 s without moving) was counted as an error, the maximal time to perform the task being 60 s. Once the platform was discovered or time had run out, the animal was allowed to rest on the platform for 15 s. Experimental groups of five to six mice were constituted and an hour rest period was allowed between each block of six trials. During the first day, the platform was alternately visible, and then hidden (2.5 cm below the water surface) for the first 12 trials. Then all trials were performed using the hidden platform configuration. For each mouse, the start location for each trial was determined randomly, while the goal arm remained constant for a given mouse. The sequence and total number of arms entered were recorded by an automatic video-tracking system (Viewpoint, Champagne au Mont d’Or, France).

### Statistics

Significant differences between groups were determined using the Kaleida Graph software. We used a one-way ANOVA followed by post hoc Fisher’s LSD or Kruskal–Wallis tests for multiple comparisons. Two-tailed unpaired Student’s *t* test was used to compare two experimental groups. Values represent the mean ± SEM of the indicated number of independent experiments/animals, and the level of significance was set for *p* < 0.05.

## Results

### Reduced amyloid burden and neuro-inflammation in the brains of TgMT5^−/−^ mice

**Fig. 1:**
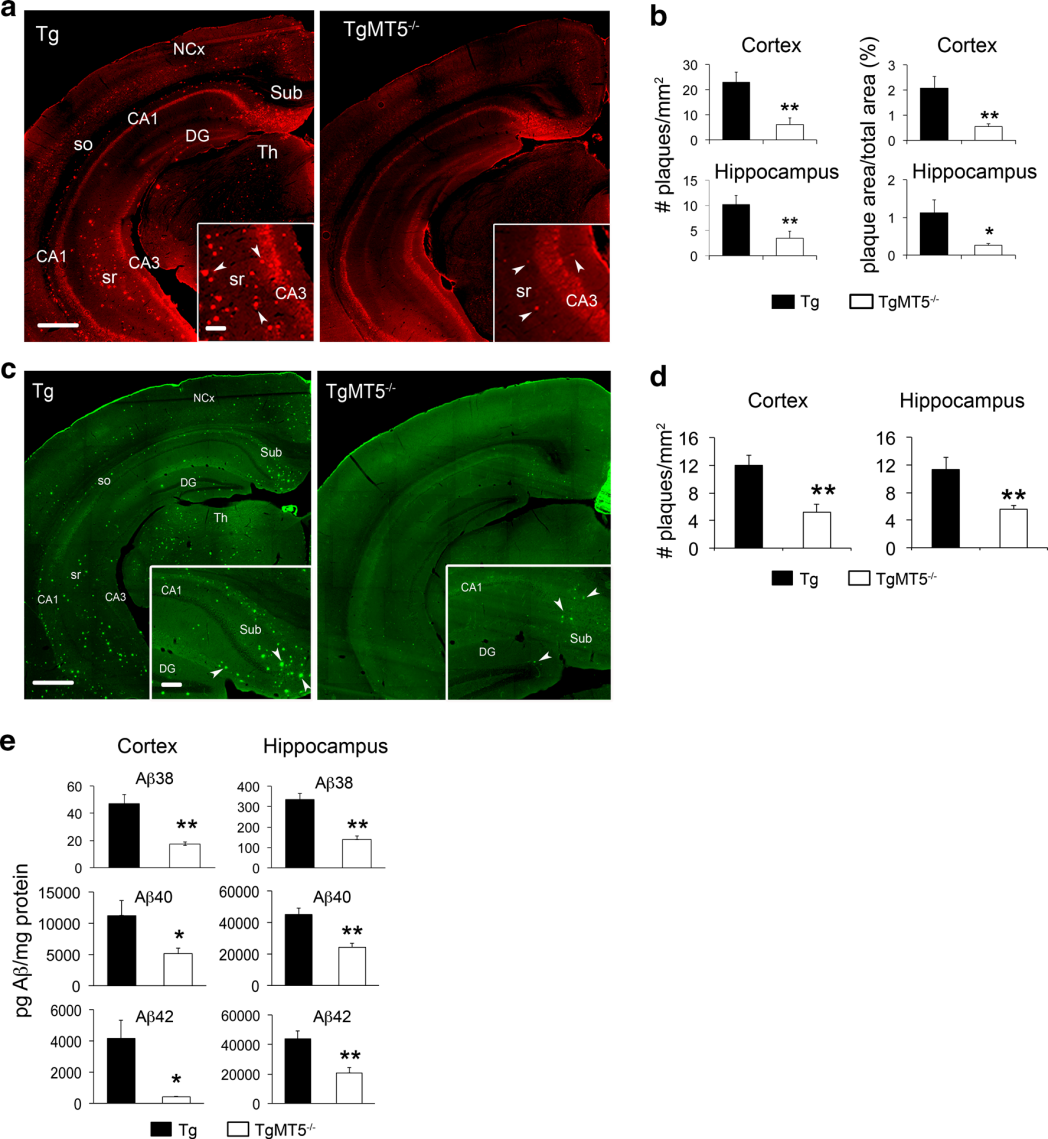
Decreased amyloid burden in the brains of TgMT5^−/−^ mice. **a** Representative epifluorescence microphotographs showing 6E10 immunohistochemistry in coronal brain sections of Tg and TgMT5^−/−^ mice. Note the dramatic reduction in the number of amyloid plaques in the cortex and hippocampus (*arrowheads* in *inset*) of TgMT5^−/−^ mice compared to Tg. *Scale bars* 500 and 100 µm. *NCx* neocortex, *Sub* subiculum, *DG* hippocampal dentate gyrus, *so* stratum oriens, *sr* stratum radiatum, *CA1-CA3* Cornu Ammonis 1 and 3 of the hippocampus, *Th* thalamus. **b** Quantification of the number of plaques and the area occupied by plaques over the total area of the indicated brain regions. **c** Representative epifluorescence microphotographs showing the reduction in amyloid plaque density between Tg and TgMT5^−/−^ brains stained with thioflavin T. *Scale bars* 500 and 100 µm. **d** Quantification of the number of thioflavin-positive plaques in the indicated brain regions. **e** ELISA assay showing a strong decrease of Aβ species in the cortex and hippocampus of TgMT5^−/−^ mice. Microphotographs are representative of seven mice per group. Values are the mean ± SEM of seven mice per group in **b** and **d**, and four mice in **e**; *p < 0.05, **p < 0.01, Student’s *t* test

We analyzed amyloid plaques in the rostral and caudal regions of the hippocampus and neocortex (somatosensory motor and visual cortices) of 4-month-old male mice using the 6E10 antibody directed against human Aβ. Consistent with previous reports [19], plaques were numerous in the subiculum and deep cortical layers of Tg mice, and more sparsely distributed in other hippocampal subfields (Fig. 1a, left). TgMT5^−/−^ mice showed a remarkable decrease in the number and total surface of amyloid plaques in the hippocampus (66 and 78 %, respectively) and neocortex (74 % for both number and total surface) (Fig. 1a right, b). Similar reductions in the number of plaques were observed after thioflavin T staining, which reveals dense core amyloid plaques (Fig. 1c, d). ELISA assay also confirmed marked reductions in soluble Aβ38 (63 %), Aβ40 (51 %) and Aββ42 (90 % in the cortex and 63 % in the hippocampus) (Fig. 1e).

**Fig. 2:**
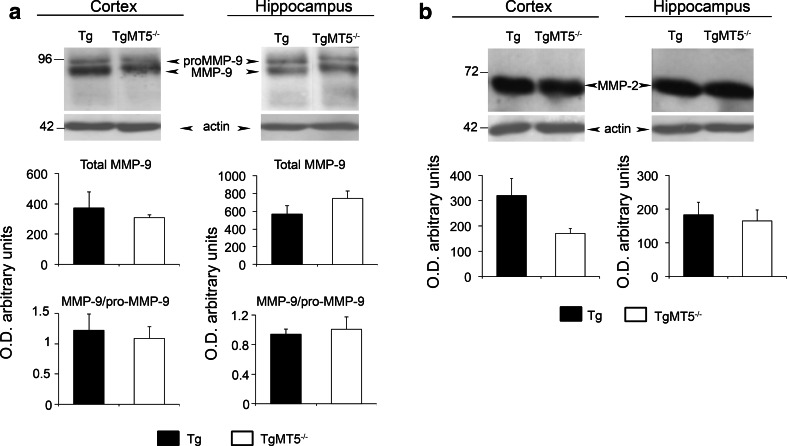
Unchanged levels of MMP-2 and MMP-9 in the brains of TgMT5^−/−^ mice. Western blots using specific antibodies and the corresponding quantifications of actin-normalized optical densities (O.D.) showing no significant differences in the levels of MMP-9 (**a**) and MMP-2 (**b**) when comparing TgMT5^−/−^ with Tg brains. Values are the mean ± SEM of six mice per group. Student’s *t* test

MMP-2 and MMP-9 are considered as the main Aβ-degrading enzymes among MMPs. Their levels in both cortex and hippocampus were not significantly altered upon MT5-MMP deficiency, though a tendency of MMP-2 to decrease was observed in the cortex (Fig. 2a, b).

**Fig. 3:**
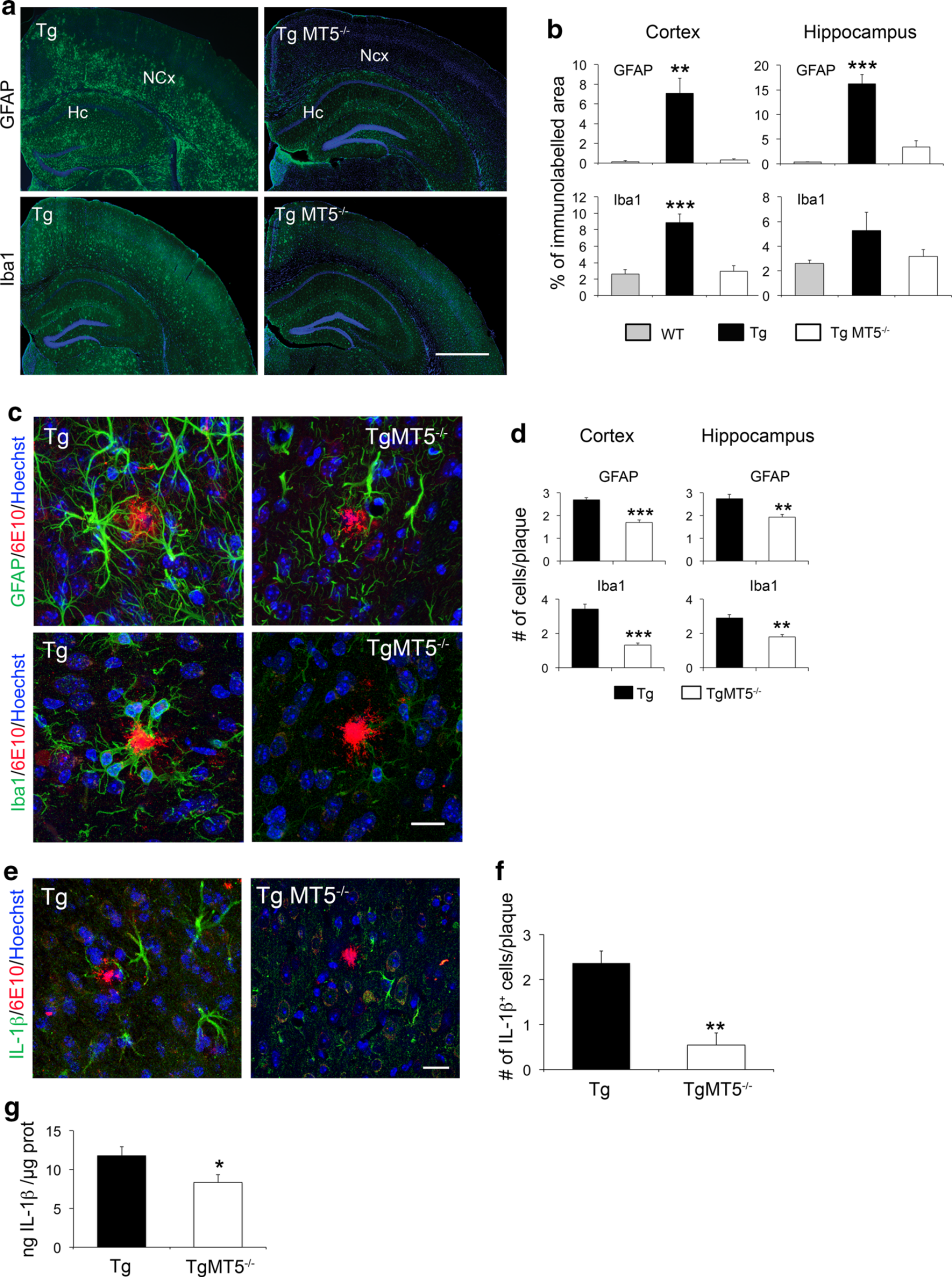
Decreased glial reactivity and IL-1β levels in the brains of TgMT5^−/−^ mice. **a** Representative epifluorescence microphotographs showing changes in astroglia (GFAP) and microglia (Iba1) reactivity in the hippocampus (Hc) and neocortex (NCx) of Tg and TgMT5^−/−^ mice, counterstained with nuclear marker Hoechst (*blue*). *Scale bar* 1 mm. **b** Quantification of changes in GFAP and Iba1 immunostaining measured as the percentage of the immunostained area/total area of the hippocampus or neocortex. Values are the mean ± SEM of seven brains per group; **p < 0.01, ***p < 0.001 vs wild type (WT) and TgMT5^−/−^ groups; ANOVA followed by post hoc Fisher’s LSD test. **c** Confocal microphotographs showing strong reductions of the density and intensity of immunoreactive astroglia (GFAP, *green*) and microglia (Iba1, *green*) around the amyloid plaque (6E10, *red*) in the neocortex of TgMT5^−/−^ compared to Tg mice. The nuclear marker Hoechst is in *blue*. *Scale bar* 20 µm. **d** Quantification of changes in the number of GFAP and Iba1-immunostained cells around plaques. Values are the mean ± SEM of seven brains per group; **p < 0.01, ***p < 0.001, Student’s *t* test. **e** Confocal microphotographs showing strong reduction of the density and intensity of immunoreactive IL-1β cells (*green*) around the amyloid plaque (6E10, *red*) in the neocortex of TgMT5^−/−^ compared to Tg mice. The nuclear marker Hoechst is in *blue*. *Scale bar* 20 µm. **f** Quantification of changes in the number of IL-1β-immunostained cells around plaques. Values are the mean ± SEM of seven brains per group; **p < 0.01, Student’s *t* test. **g** ELISA assay showing significant decrease of IL-1β levels in the cortex of TgMT5^−/−^ mice. Values are the mean ± SEM of seven to nine mice per group; *p < 0.05, Student’s *t* test

Glial reaction and inflammatory processes are systematically associated with Aβ deposition, although their precise role in AD pathogenesis remains elusive [35]. The 5xFAD AD model is not an exception and gliosis increases across time with the progression of pathology [19, 36]. As illustrated in Fig. 3a, b, TgMT5^−/−^ mice exhibited strong reductions of astroglial (GFAP) inmmunostaining in the cortex (90 %) and hippocampus (70 %). Microglial staining (Iba1) was only significantly decreased in the cortex (79 %). Also, the number of GFAP^+^-reactive astrocytes around amyloid plaques decreased in the neocortex (−38 %) and hippocampus (−30 %) of TgMT5^−/−^ mice, while Iba1^+^ microglial cells decreased by 67 and 47 % in the same areas (Fig. 3c, d). Tg brains exhibited reactive microglia characterized by hypertrophic extensions mingled with amyloid plaques, in clear contrast with TgMT5^−/−^ microglia, which displayed much thinner extensions only occasionally associated with plaques (Fig. 3c). There was further evidence of diminished inflammatory response in the TgMT5^−/−^ brains steming from decreased levels of IL-1β, which stands among the most potent mediators of inflammation. Indeed, the number of IL-1β-immunostained cells around amyloid plaques was dramatically reduced by threefold in TgMT5^−/−^ brains with respect to Tg brains (Fig. 3e, f), and the levels of the cytokine dropped by 30 %, as measured by ELISA assay (Fig. 3g). Double labeling experiments confirmed that all IL-1β cells were also immunoreactive for the astrocyte marker GFAP (Online resource 2). Overall, MT5-MMP deficiency was clearly associated with a reduction of both amyloidosis and neuroinflammation.

### Decreased C-terminal APP fragments and stable α-, β- and γ-secretase activities in the brains of TgMT5^−/−^ mice

**Fig. 4:**
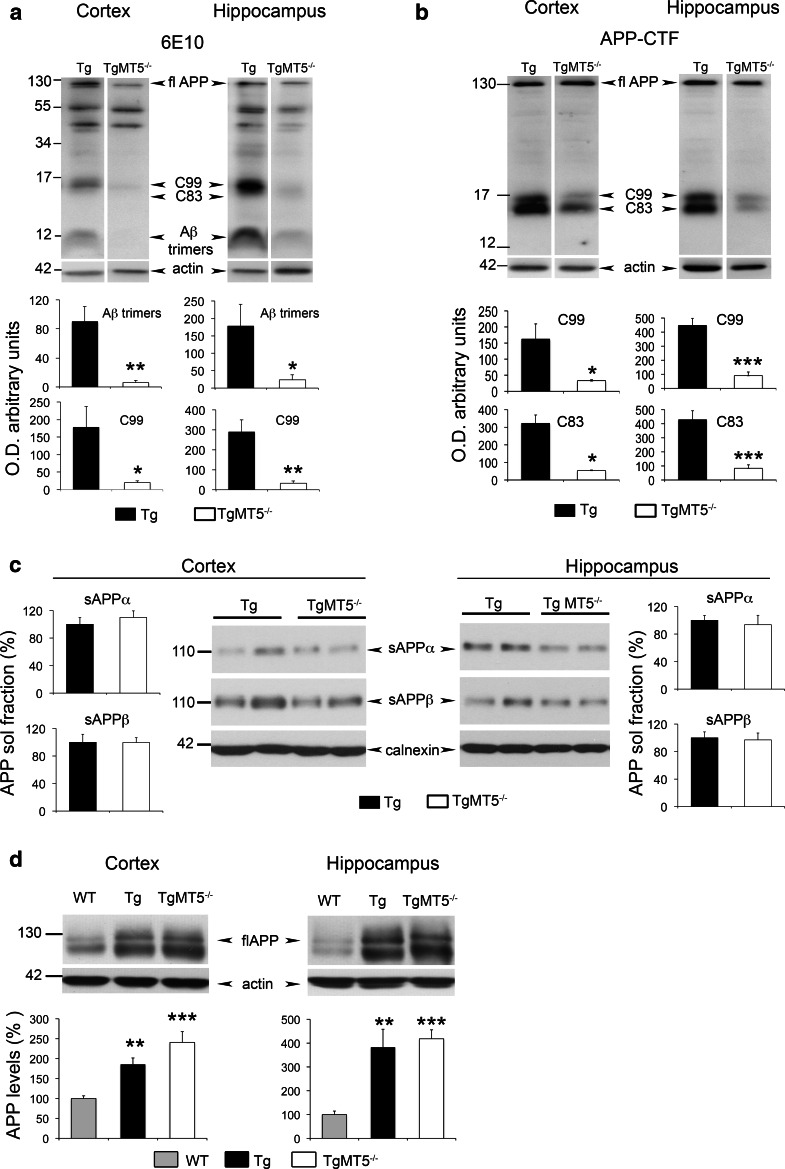
Decreased Aβ small oligomers and α- and β-CTFs in the brains of TgMT5^−/−^ mice. **a** Western blot using the 6E10 antibody showing decreased levels of Aβ trimers and β-CTF (C99) in the cortical and hippocampal insoluble fractions of TgMT5^−/−^ mice compared to Tg. Quantifications of actin-normalized optical densities (O.D.) are presented below. *fl* full length. **b** Western blot using the APP-CTF antibody showing decreased levels of β- and α-CTFs, C99 and C83, respectively. Quantifications of actin-normalized optical densities (O.D.) are presented below. **c** Western blot using specific 14D6 and 192swe antibodies and the corresponding quantifications showing no changes in sAPPα and sAPPβ calnexin-normalized levels in cortical and hippocampal soluble fractions of TgMT5^−/−^ and Tg mice. **d** Western blot and the corresponding quantifications of full-length (*fl*) APP levels using the 22C11 antibody on whole tissue homogenates, showing increased APP levels in transgenic mice with respect to WT, but no changes between Tg and TgMT5^−/−^. Values are the mean ± SEM of seven to nine mice per group; *p < 0.05, **p < 0.01, ***p < 0.001, Student’s *t* test for **a**, **b** and **c**. ANOVA followed by post hoc Fisher’s LSD test for **d**

**Fig. 5:**
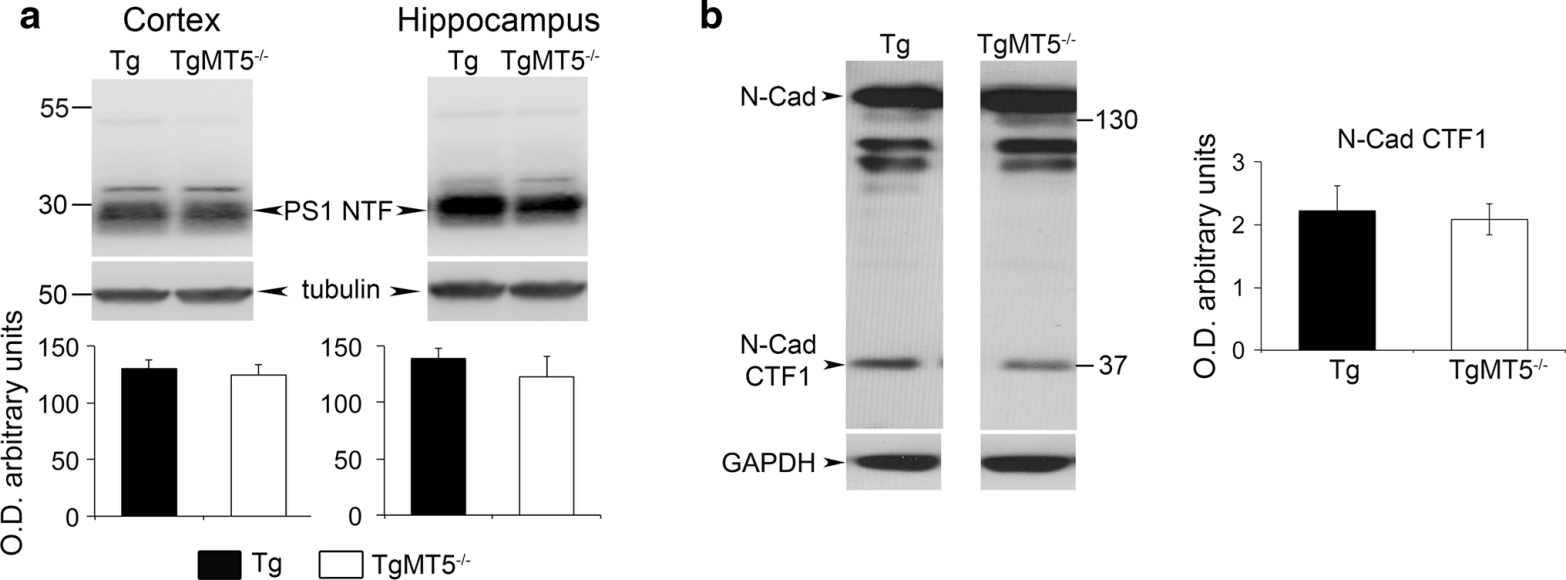
Unaltered γ-secretase activity in the brains of TgMT5^−/−^ mice. **a** Representative western blots of PS1-NTF (N-terminal fragment) content resulting from PS1 autocatalysis in cortical and hippocampal solubilized membranes. The corresponding tubulin-normalized quantifications below show no differences between Tg and TgMT5^−/−^ mice. **b** Representative western blot of N-cadherin-immunoreactive bands and the corresponding GAPDH-normalized quantifications showing stable levels of γ-secretase substrate N-cadherin C-terminal fragment 1 (CTF1) in the hippocampal insoluble fraction of TgMT5^−/−^ compared to Tg. Normalized values are the mean ± SEM of seven to eight brains per group, Student’s *t* test

Small Aβ oligomers are increasingly considered as the main triggers of neurodegeneration and cognitive decline in AD [37–39]. Using the 6E10 antibody, which recognizes an epitope located between the α- and β-sites of the Aβ/C99 sequence, we found in the insoluble fraction of TgMT5^−/−^ cortex and hippocampus a 90 % reduction of a 12 kDa immunoreactive band that could correspond to Aβ trimers (Fig. 4a). No changes, however, were detected in 6E10-immunoreactive bands of higher molecular weights (e.g., ~44, ~56 kDa) (Fig. 4a). It is noteworthy that the 12 kDa band was not detected by the APP-CTF antibody, which recognizes the C-terminal end of APP (Fig. 4b). Together, these data support the idea that the 12 kDa band likely represents Aβ trimers (Fig. 4a). However, we cannot rule out the possibility that it corresponds to Aβ fragments N-terminally elongated. These could result from the combined MT5-MMP cleavage on APP residues 504–505 upstream from the α- and β-site of cleavage, as previously suggested [11], and α-secretase-mediated cleavage. This putative ~12 kDa fragment would preserve the epitope recognized by the 6E10 antibody. MT5-MMP deficiency was also accompanied by drastic reductions of a doublet band below 17 kDa recognized by the APP-CTF antibody (Fig. 4b). The 6E10 antibody only recognized the upper band of the doublet (Fig. 4a), suggesting that the doublet features C99 and C83, the β- and α-secretase-derived APP C-terminal fragments, respectively. The levels of the N-terminal counterparts of hC83 and hC99, namely, hsAPPα and hsAPPβ, can be specifically and, respectively, monitored by the 14D6 and 192swe antibodies, which are, respectively, considered as a readout of α- and β-secretase activities [2]. We found no differences in the levels of these soluble APP fragments when comparing TgMT5^−/−^ to Tg brains (Fig. 4c). As expected, the expression of the human *APP* gene increased the cortical and hippocampal levels of full-length APP in Tg and TgMT5^−/−^ compared to WT mice brains, but no changes were observed between Tg and TgMT5^−/−^ (Fig. 4d). Finally, we investigated a possible alteration of the γ-secretase complex and found no differences in the levels of the ~30 kDa PS1 N-terminal fragment (PS1-NTF) (Fig. 5a); the latter results from autocatalysis of PS1 and reflects biologically active conformation of the γ-secretase complex [40]. Seemingly, the content of other members of the γ-secretase complex (Aph1, Pen2 and nicastrin) remained unaltered in TgMT5^−/−^ mice (Online resource 3). The γ-secretase complex processes numerous type I transmembrane proteins, including cadherins [41]. γ-secretase can further cleave the C-terminal 37 kDa fragment (CTF1) of N-cadherin into CTF2, leading to the accumulation of CTF1 upon γ-secretase inhibition [42, 43]. We found that CTF1 content was not affected in TgMT5^−/−^ hippocampus (insoluble fraction) compared to Tg (Fig. 5b), further supporting normal function of the γ-secretase complex.

### Long-lasting beneficial effects of MT5-MMP deficiency at advanced stages of the pathology

**Fig. 6:**
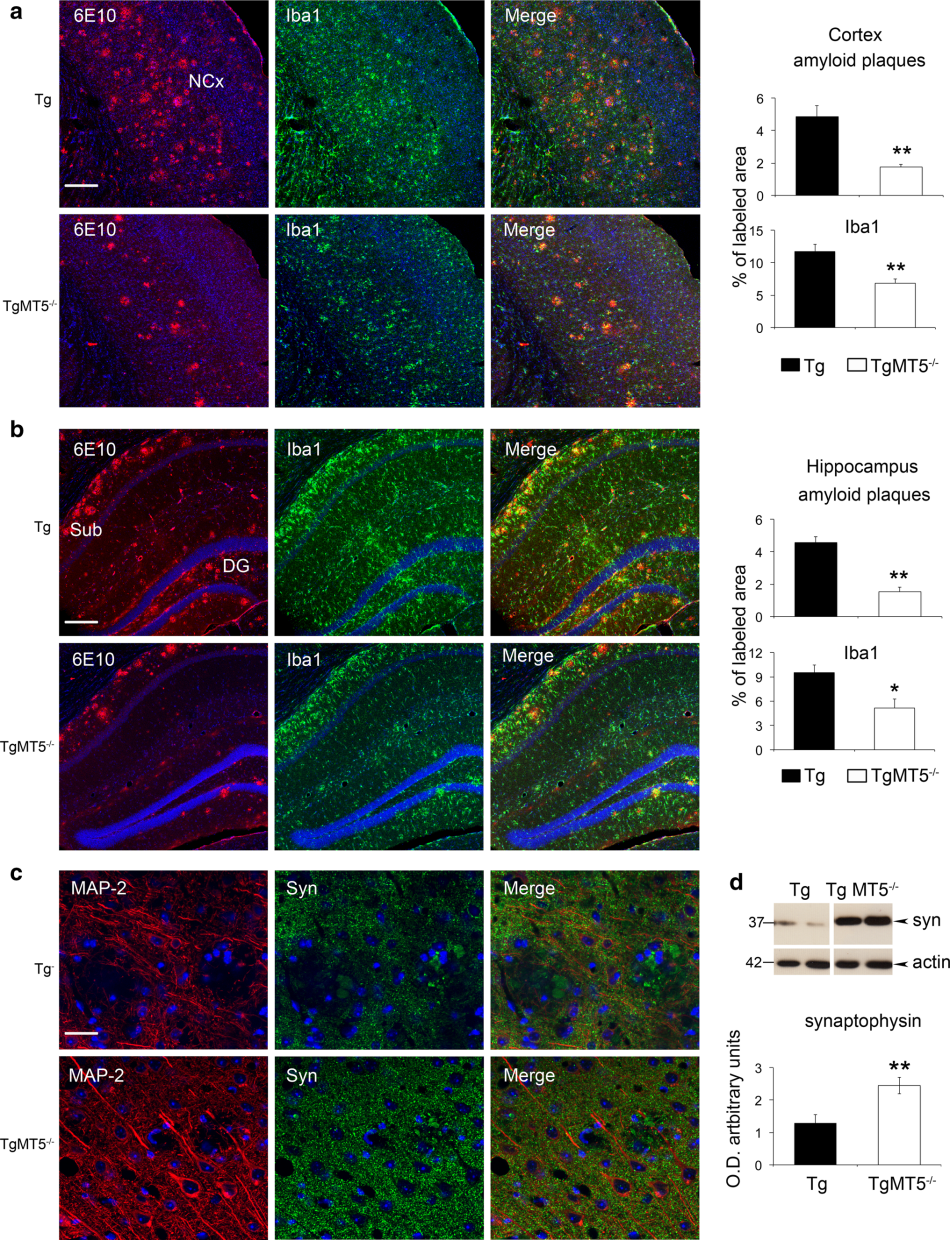
Long-lasting beneficial effects of MT5-MMP deficiency in the brains of TgMT5^−/−^ mice. Representative confocal microphotographs of seven mice per group and the corresponding quantifications showing strong reductions in plaque load (6E10, *red*) and microglial reactivity (Iba1, *green*) in the neocortex (**a**) and hippocampus (**b**) of 16-month-old TgMT5^−/−^ mice compared to age-matched Tg mice. The nuclear marker Hoechst is in *blue*. *Scale bar* 200 µm. *NCx* neocortex, *Sub* subiculum, *DG* dentate gyrus. **c** Representative confocal microphotographs showing a better-preserved neuronal network (MAP-2, *red*) and increased synaptophysin immunostaining (Syn, *green*) in layers IV–V of the neocortex. *Scale bar* 20 µm. **d** Western blot of cortical soluble fractions showing increased levels of synaptophysin in TgMT5^−/−^ mice compared to Tg. Values are the mean ± SEM of seven actin-normalized optical densities (O.D.); **p < 0.01, Student’s *t* test

Synaptotoxicity is one of the pathological signs that accompany Aβ accumulation in 5xFAD mice and the associated deficits of learning and emotional behaviors at advanced stages of the pathology [44, 45]. To analyze a potentially durable positive impact of MT5-MMP deficiency, we evaluated amyloid burden and neuronal network integrity at 16 months of age in the neocortex (somatosensory motor and visual cortices) and hippocampus. The content of amyloid plaques was reduced in both areas of TgMT5^−/−^ mice by 64 % and 66 %, respectively (Fig. 6a, b). Concomitantly, microglial reactivity dropped by 42 and 46 % in the same areas (Fig. 6a, b). In good correlation with lower plaque density and gliosis, TgMT5^−/−^ mice exhibited a better preservation of the neuronal network, as illustrated by MAP-2 immunostaining (Fig. 6c) and by 90 % higher levels of cortical synaptophysin (Fig. 6d) when compared with Tg mice. Overall, relevant long-lasting beneficial effects of MT5-MMP deficiency were still present at advanced stages of the pathology.

### MT5-MMP interacts with APP, stimulates Aβ and C99 production and releases a soluble APP fragment of 95 kDa

**Fig. 7:**
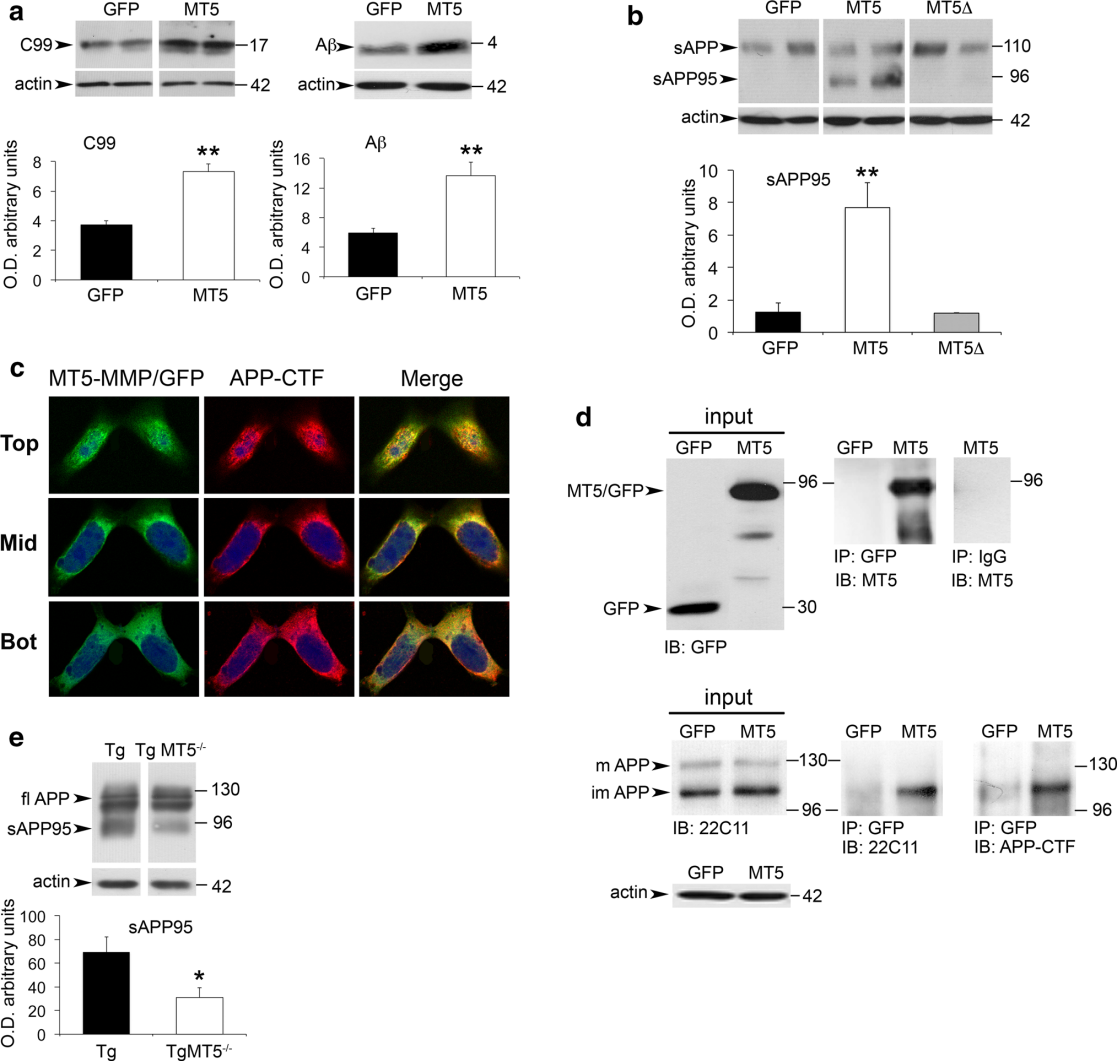
MT5-MMP increases C99 and Aβ, interacts with APP and promotes the release of a 95 kDa sAPP fragment in vitro and in vivo. **a** Western blots and the corresponding quantifications showing strong increases of C99 and Aβ levels in HEKswe lysates and supernatants after transfection of MT5-MMP/GFP (MT5) and GFP plasmids. Values are the mean ± SEM of six independent experiments; **p < 0.01, Student’s *t* test. **b** Western blots and the corresponding quantifications showing the generation of a 95 kDa sAPP fragment (sAPP95) in the supernatants of HEKswe transfected with MT5-MMP/GFP (MT5), but not with its mutated inactive variant MT5-MMPΔ/GFP (MT5Δ). Values are the mean ± SEM of six independent experiments. **p < 0.01, ANOVA followed by post hoc Fisher’s LSD test. **c** Confocal microphotographs representative of six independent cultures in HEKswe cells showing partial co-localization (*yellow* *spots*, merge) of MT5-MMP and APP 48 h after transfection of MT5-MMP/GFP at the *top*, *middle* and *bottom* cell levels using anti-GFP and APP-CTF antibodies. **d** Western blots representative of four independent cultures showing the input and the immunoprecipitation (IP) of MT5-MMP/GFP and APP in lysates of HEKswe cells 48 h after transfection with GFP control or MT5-MMP/GFP (MT5) plasmid. *Upper panel* input and IP for MT5-MMP/GFP revealed by immunoblot (IB) with anti-GFP and anti-MT5-MMP antibodies, respectively. All the IP experiments were performed with a mouse anti-GFP antibody. Negative IP control using mouse anti-IgG is represented on the *right* column. *Lower panel* input and IP of full-length putative mature (*m*) and immature (*im*) APP forms. Note that only the lower APP form is immunoprecipitated, as revealed by IB with 22C11 (N-ter) and APP-CTF (C-ter) antibodies. Actin was used as a loading control. **e** Western blots using the 22C11 antibody and the corresponding quantifications showing a strong decrease of sAPP95 levels in the soluble fractions of hippocampal homogenates from TgMT5^−/−^ mice compared to Tg mice. Values are the mean ± SEM of actin-normalized optical densities (O.D.) from 6 mice per group; *p < 0.05, Student’s *t* test

The in vivo data suggested that MT5-MMP could promote amyloidosis. This contingency was further supported in culture experiments using genetically engineered HEKswe cells that harbor one of the amyloidogenic APP mutations carried by 5xFAD mice. We found that overexpressing in these cells MT5-MMP fused to a GFP tag induced a 2-fold increase of C99 in cell lysates and a 2.5-fold increase of Aβ levels in cell supernatants, compared with control cells transfected with GFP plasmid (Fig. 7a). In the same experimental setting, we observed that MT5-MMP induced the release of a truncated soluble sAPP fragment of 95 kDa. This sAPP95 soluble fragment was not generated in cells transfected with the control plasmid or the mutated catalytically inactive MT5-MMP (MT5Δ) variant (Fig. 7b), clearly indicating that the production of sAPP95 required the proteolytic activity of MT5-MMP.

These data, together with previous reports indicating the processing of APP by MT5-MMP [11], led us to hypothesize a possible interaction between MT5-MMP and APP not previously reported. The hypothesis was supported by two experimental evidences: (1) double immunocytochemistry experiments revealed partial colocalization of MT5-MMP and APP in MT5-MMP-transfected cells in perimembranous and cytosolic compartments (Fig. 7c); (2) MT5-MMP/GFP immunoprecipitated endogenous APP (Fig. 7d). Moreover, APP-CTF antibody immunoprecipitated MT5-MMP/GFP (Online resource 4a), but did not immunoprecipitate overexpressed TfR/GFP, which was used as negative control. As additional control of specific MT5-MMP/APP interactions, we showed that MT5-MMP/GFP did not immunoprecipitate the endogenous or overexpressed TfR (Online resource 4b). Altogether, these data provide the first evidence of MT5-MMP/APP interactions. To explore whether MT5-MMP deficiency could also affect putative sAPP95 levels in vivo, we analyzed the N-terminal fragments of APP using the 22C11 antibody. A fragment of 95 kDa was indeed detected in the soluble fraction of Tg brains at 2 months of age, and most interestingly its levels were reduced in the cortex (51 %, not shown) and hippocampus (55 %) of TgMT5^−/−^ mice compared to Tg (Fig. 7e).

### Preserved LTP in the hippocampus of TgMT5^−/−^ mice

**Fig. 8:**
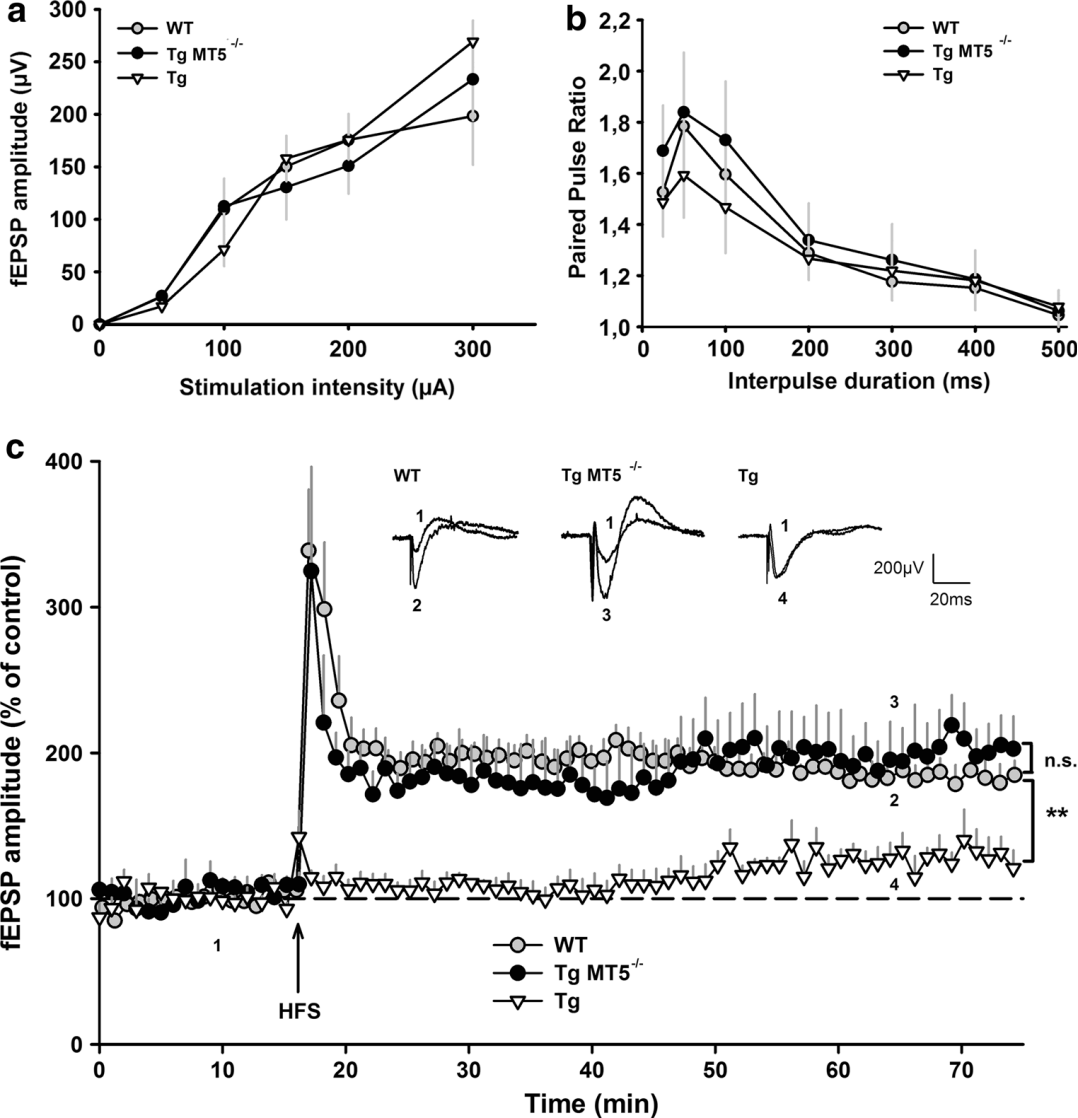
Preserved LTP in the hippocampus of TgMT5^−/−^ mice. **a** Properties of the excitatory synaptic transmission in the hippocampus of WT, TgMT5^−/−^ and Tg mice. Basal synaptic transmission was estimated by generating input–output (I/O) curves and by plotting the amplitude of field excitatory postsynaptic potentials (fEPSPs) versus the stimulation intensity applied to the Schaffer collaterals pathway. **b** Short-term synaptic plasticity was assessed by eliciting paired-pulse facilitation. Paired-pulse facilitation ratio was calculated by dividing the amplitude of the second fEPSP by the amplitude of the first one. **c** Long-term potentiation (LTP) was obtained by applying brief high-frequency stimulation (HFS: 100 Hz, 1 s). HFS triggered LTP in both WT (*gray circles*, *n* = 6) and TgMT5^−/−^ mice (*black circles*, *n* = 6), but no LTP was observed in Tg mice 45 min after HFS (*white triangles*, *n* = 6). Representative traces of fEPSP are shown above the LTP time course. They have been extracted at the indicated time points for WT (1 and 2), TgMT5^−/−^ (1 and 3) and Tg (1 and 4) mice. Values are the mean ± SEM of six mice per group. ** *p* < 0.01 and nonsignificant (*n.s.*), on comparing data obtained from Tg and TgMT5^−/−^ groups with those obtained from the WT group; ANOVA followed by Kruskal–Wallis test

We next examined if MT5-MMP deficiency could influence synaptic functions. I/O curves of fEPSPs in response to different stimulation strengths were not different across WT, Tg and TgMT5^−/−^ mice, indicating similar basal synaptic transmission (Fig. 8a). Paired-pulse facilitation represents a short-term form of synaptic plasticity and the probability of presynaptic glutamate release; this parameter was also unaffected across genotypes (Fig. 8b). Finally, we examined LTP (Fig. 8c), considered as the cellular substrate of learning and memory processes [46]. A classic stimulation protocol (HFS: 100 Hz, 1 s) was sufficient to induce robust LTP in slices from 4-month-old WT and TgMT5^−/−^ mice for more than 50 min, which reached 187 and 195 % of the basal fEPSP amplitude, respectively.

This protocol unveiled a deficiency in LTP in Tg mice at 4 months not previously detected using theta burst protocols [47]. Indeed, CA1 LTP in Tg hipppocampi reached only 127 % of the basal fEPSP amplitude 50 min after HFS delivery (Fig. 8c). This weak LTP in Tg mice was in clear contrast with robust LTP exhibited by TgMT5^−/−^ mice (195 %), indicating that MT5-MMP deficiency contributed to LTP preservation (Fig. 8c).

### Improved performance of TgMT5^−/−^ mice in the six-arm radial water maze

**Fig. 9:**
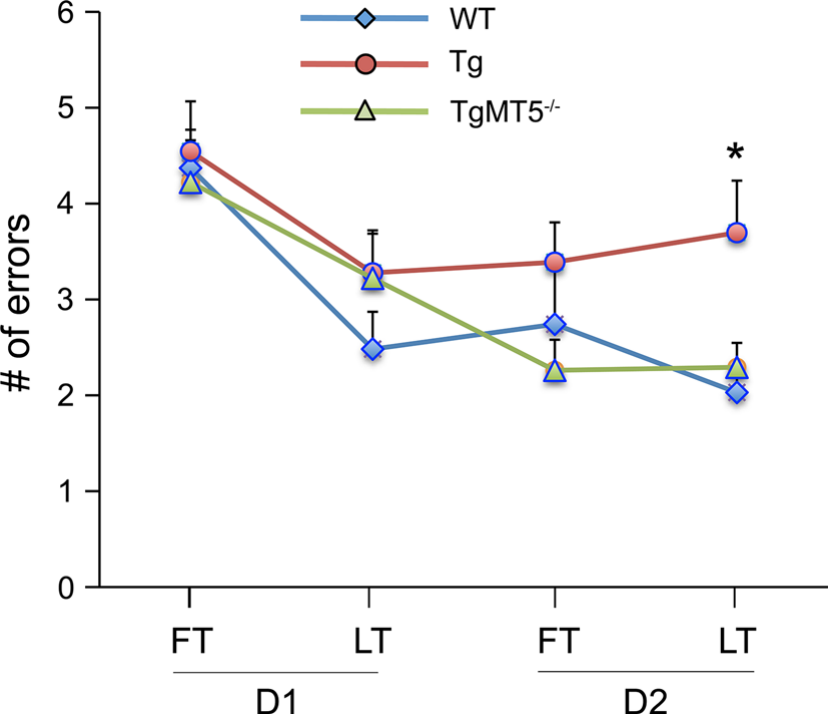
Improved hippocampal-dependent associative spatial learning in TgMT5^−/−^ mice compared to Tg mice. Graph representing 6-ARWM task in* WT*,* Tg* and* TgMT5* ^−/−^ mice. The number of errors made by mice in the first (*FT*) and last (*LT*) trials of day session 1 (D1) or 2 (D2) was evaluated as a measure of learning consolidation. Like *WT*,* TgMT5* ^−/−^ mice show a decrease in the number of errors that is significantly different from Tg mice by the end of the test. Values represent the mean ± SEM of 10–12 mice per group * *p* < 0.05, on comparing* Tg* to* WT* and* TgMT5* ^−/−^ groups; ANOVA followed by post hoc Fisher’s LSD test

The 6-ARWM [34], conceived to improve the sensitivity of the Morris Water Maze test, was used to study spatial memory in our mice. We found that WT and TgMT5^−/−^ mice made significantly less errors than Tg mice by the end of the 2-day learning period, highlighting a preservation of spatial learning and memory in TgMT5^−/−^ mice (Fig. 9).

## Discussion

MT5-MMP is the only MMP predominantly expressed in the nervous system. Despite growing importance of MT5-MMP in nervous function and disease, and some evidence indicating functional interactions with key elements of the amyloidogenic process, its role in Alzheimer’s pathology remains unexplored. Accordingly, we have tested the hypothesis that MT5-MMP may influence amyloid-associated pathology and provide the first compelling evidence that this is the case in the 5xFAD mouse model of AD. We show in early stages of the pathology that MT5-MMP deficiency dramatically reduces amyloid load and C99 levels in the cortex and hippocampus of 5xFAD mice, which were concomitant with reduced neuroinflammation and the preservation of LTP and cognitive functions in bigenic mice. Long-lasting beneficial effects of MT5-MMP deficiency were also observed at advanced stages of the pathology, further highlighting the importance of MT5-MMP in the control of amyloidosis and associated neurodegeneration. Moreover, we discovered physical interactions between MT5-MMP and APP, and provide convergent data involving MT5-MMP in the processing of APP in vitro and in vivo. Together, the functional interactions between MT5-MMP and APP, without changes in the activity of the main secretases, may indicate that MT5-MMP acts somewhere along the amyloidogenic pathway to modulate APP trafficking/bioavailability and subsequently its processing.

The striking effects of MT5-MMP deficiency on Aβ load are reminiscent of those observed upon *BACE1* deletion [48], or conditional PS1 knockout [49]. Yet, the putative APP cleavage of MT5-MMP (and also MT1-MMP and MT3-MMP) of about 100 residues N-terminal upstream from the β-cleavage site [11] suggests that the MMP does not harbor a β-secretase-like activity, which could directly initiate Aβ generation. The stimulating effect of MT5-MMP on amyloidosis throughout our in vivo and in vitro experiments is in keeping with recent findings uncovering pro-amyloidogenic properties of MT1-MMP [9]. It is thus possible that the MT5-MMP and MT1-MMP process APP in a similar manner. The degree of functional redundancy or specificity between these MMPs and how this affects AD pathogenesis remains to be determined. In any case, the pro-amyloidogenic features of MT5-MMP are in clear contrast with the Aβ-degrading activities classically attributed to soluble MMPs, i.e., MMP-2 and MMP-9 [9, 50, 51]. Such divergence could represent a fundamental difference in the way soluble and membrane-bound MMPs interact with APP/Aβ and contribute to their processing.

Simultaneously to reduced Aβ burden in TgMT5^−/−^ brains, the decrease of glial reactivity and IL-1β levels illustrate a diminished inflammatory response in bigenic mice. Prominent IL-1β expression in reactive astrocytes of Tg mice is in agreement with similar astrocyte-restricted IL-1β expression found around amyloid plaques early after exogenous injection of Aβ1-42 [52]. From a functional standpoint, the inhibition of IL-1β signaling has been reported to partly reduce fibrillar and oligomeric Aβ and to improve cognition in a mouse model of AD [53]. It is also noteworthy that MT5-MMP deficiency alters IL-1β signaling in the peripheral nervous system in a non-AD context [16]. Taken together, these findings advocate: (1) a role of astrocytes as key mediators of the Aβ-driven inflammatory response in early phases of the pathological process; and (2) a possible functional link between MT5-MMP and IL-1β-mediated inflammation, whereby MT5-MMP deficiency could interfere with IL-1β signaling. It is therefore plausible that a reduction in Aβ and IL-1β levels would jointly contribute to tune down microglial activation and homing around plaques, as observed in TgMT5^−/−^ brains.

Small Aβ oligomers, including dimers and trimers, appear to be important in vivo Aβ assemblies [54], with neurotoxic properties that undermine memory [55, 56] and LTP [38, 57]. Accordingly, the remarkable reductions of Aβ trimers exhibited by TgMT5^−/−^ brains correlate with the positive outcome of the pathology, possibly indicating a causal effect. Additional beneficial effects for TgMT5^−/−^ mice may result from the strong reduction in the levels of Aβ precursor C99, which has been shown to be neurotoxic on its own [58]. Interestingly, we have previously observed abnormal cortico-hippocampal accumulation of C99 in 5xFAD mice as early as 2 months of age, whereas Aβ trimers are first detected at 4 months [9]. Drastic reductions in C99 upon MT5-MMP deficiency should be in principle coherent with changes in canonical APP or in the enzymatic systems that contribute to its generation (β-secretase) or conversion into Aβ (γ-secretase). However, none of these possibilities was supported by our data, which indicated stable levels of full-length APP and β-secretase activity between Tg and TgMT5^−/−^ brains. Furthermore, unaltered PS1 autocatalysis or processing of the N-cadherin CTF1 fragment supported normal γ-secretase activity in TgMT5^−/−^ brains, as well. This is in good agreement with the reported finding that another important substrate of γ-secretase, Notch, remains stable in MT5-MMP-deficient mice [15]. In the absence of noticeable secretase dysfunctions, the differential impact of MT5-MMP deficiency on soluble and intracellular APP fragments suggest that MT5-MMP may influence the intracellular trafficking of APP and/or its targeting to subcellular compartments where C99 is degraded. Even though this hypothesis needs to be further investigated, it is consistent with previous reports showing that proteasome/lysosome activity accounted for significant degradation of C99 [59, 60]. Most interestingly, these studies suggested that decreases in C99 levels could be independent of γ-secretase activity, thus providing with alternative mechanisms to interpret changes in C99 turnover. Our experiments showing MT5-MMP/APP interactions and colocalization in perimembranous and intracellular compartments of HEKswe further support the idea that MT5-MMP could be involved in APP trafficking.

It is also noteworthy that MT5-MMP induces the release of a truncated APP fragment of 95 kDa, which is reminiscent of the N-terminal APP fragment reported in HEK_WT_ cells transfected with either MT1-, MT3- or MT5-MMPs [11]. In this study, the putative MT-MMP cleavage site was identified between residues 504–505 of recombinant APP. Our findings in HEKswe cells corroborate these previous observations in HEK_WT_ cells, but most importantly provide the first in vivo evidence that the levels of a 95 kDa APP N-terminal fragment are reduced in TgMT5^−/−^ brains compared to Tg. Altogether, these data support the idea that MT5-MMP can directly or indirectly influence APP processing in vivo. Wether there is a functional link between the MT5-MMP-mediated proteolytic event that releases sAPP95 and amyloidosis remains to be elucidated. In this context, one could expect that MT5-MMP activity generates a residual C-terminal APP fragment of ~25 kDa, but such fragment was not consistently detected in our experimental conditions, suggesting high instability. In the same vein, an unprecedentedly described APP C-terminal fragment of 25 kDa—named eta-CTF (η-CTF)—has been recently reported in various cell lines only after treatment with inhibitors of protein degradation [61]. Interestingly, the generation of η-CTF is independent of BACE1 activity and is prevented by MMP inhibitors (A. Saunders, personal communication). Whether MT5-MMP or other MT-MMPs contribute to η-CTF production requires further investigation. Overall, gaining knowledge on alternative proteolytic APP processing should eventually help us to better understand the fate of about 50 % of APP processing that is not explained merely by the action of β-secretase, as reported [62].

In agreement with previous studies on 5xFAD mice [47] AMPA/kainate (GluA) receptor-mediated synaptic responses (basal synaptic transmission) and short-term presynaptic plasticity (paired-pulse facilitation) were not affected in our Tg or TgMT5^−/−^ mice. This implies that MT5-MMP deficiency in vivo does not interfere with GluA functioning, as could be possibly inferred from functional interactions reported between MT5-MMP and GluA receptors in hippocampal neuronal cultures [63]. Thus, the remarkable preservation of LTP in TgMT5^−/−^ mice compared to Tg likely results from the robust reduction of Aβ burden, and in particular of Aβ trimers, which are considered to be powerful inhibitors of LTP [38]. Overall, small Aβ oligomers are increasingly seen as a major trigger of synaptic dysfunction in AD [64] by altering NMDA receptors containing the GluN2B subunit [65]. A plausible functional link between reduced amyloidosis and preserved synaptic plasticity is further supported by the improvement of LTP observed in bigenic 5xFAD/BACE1^+/−^ mice, where strong decreases in Aβ levels are comparable to those found in our TgMT5^−/−^ mice [66].

Using the 6-ARWM, we showed for the first time mild impairment of hippocampal-dependent associative spatial learning in 5xFAD mice at 4 months of age, in agreement with previous studies reporting that the first signs of behavioral disruption in 5xFAD mice occur between 4 and 5 months of age [19, 36]. Preliminary experiments failed to demonstrate significant alterations of learning using the classic Morris Water Maze test. This stresses the importance of using suitable tests to decipher subtle learning deficits characteristic of early phases of the degenerative process. Most importantly, we demonstrate that TgMT5^−/−^ mice perform better than Tg mice in a context of preserved LTP. Similar positive correlations between learning and LTP have been reported in other AD mouse models, where 50 % reduction in Aβ levels was sufficient to improve LTP and spatial learning [67]. Altogether, the age of 4 months in 5xFAD mice may represent a “prodromal-like” stage of the pathology, where we are able to detect subtle alterations of behavior. This highlights the potential therapeutic interest of modulating MT5-MMP activity before irreversible damage occurs.

We conclude that brain MT5-MMP is endowed with a regulatory role on processes that influence pathogenesis and cognitive decline in AD. MT5-MMP emerges as a proteinase that seems to exert multiple regulatory effects on APP metabolism, possibly mediated in a non-exclusive manner by protein–protein interactions and/or proteolytic processing. Further investigation will be required to gain insight into the nature of these interactions to better understand how MT5-MMP deficiency keeps Aβ load within a “safe” range in a durable manner without impacting on the activity of β- and γ-secretases. This could be of therapeutic relevance in the prospects of designing MT5-MMP-based strategies to modulate rather than inhibit secretase activities. Our data support the idea that MMPs are not only Aβ degrading enzymes and hence potentially beneficial, but on the contrary, some MMPs may promote Aβ/C99 accumulation and neurodegeneration. For these reasons, caution should be observed when targeting MMP activity in a non-specific manner. Accordingly, this study should lay the basis to encourage the design of therapeutic strategies to specifically modulate MT5-MMP activity and/or interactions with APP. Moreover, MT5-MMP expression being essentially restricted to neural tissues may further highlight the relevance of this proteinase as a therapeutic target to alleviate amyloid-mediated pathology in AD.

### Electronic supplementary material

Below is the link to the electronic supplementary material.

**Online resource 1**. Efficient knock out of MT5-MMP in TgMT5^−/−^ brains. Western blot using our own developed specific rabbit anti-mouse polyclonal MT5-MMP antibody in homogenates of hippocampus and cerebellum from P15 Tg and TgMT5^−/−^ mice showing efficient knock out of MT5-MMP (TIFF 628 kb)

**Online resource 2**. Astrocytic expression of IL-1β and strong reduction of IL-1β immunostaining in TgMT5^−/−^ astrocytes. Confocal microphotographs representative of 7-8 mice per group showing double immunostained cortical sections with co-localization (yellow-arrowheads) of IL-1β (green) and GFAP (red) in the neocortex of Tg and TgMT5^−/−^ mice. Note also the strong reduction in IL-1β immunostaining in TgMT5^−/−^ brains compared to Tg. Scale bar : 20 µm (TIFF 8418 kb)

**Online resource 3**. Expression of γ-secretase members Aph1, Pen-2 and nicastrin in the brains of Tg and TgMT5^−/−^ mice. Representative western blots of Aph1, Pen-2 and nicastrin (Nct) in cortical and hippocampal homogenates, showing no differences between TgMT5^−/−^ and Tg brains. Values represent the mean ± SEM of tubulin normalized optical densities (O.D.) from 7-8 brains per group (TIFF 380 kb)

**Online resource 4**. MT5-MMP/GFP and APP do not interact with endogenous or overexpressed transferrin receptor. a. Western blots representative of 4 independent cultures showing that immunoprecipitated APP interacts with MT5-MMP/GFP, but not with the overexpressed transferrin receptor (TfR) in lysates of cultured HEKswe cells 48 h after transfection with GFP control, MT5-MMP/GFP (MT5) or TfR/GFP (TfR) plasmids. Upper panel: input for overexpressed plasmids immunoblotted (IB) with anti-GFP antibodies. Lower panel: immunoprecipitation (IP) of endogenous APP with APP-CTF antibodies and IB with anti-GFP antibodies. Actin loading controls are representative of all inputs in a and b. **b**. Western blots repesentative of 4 independent cultures showing that MT5-MMP/GFP does not interact with TfR in the same experimental conditions described in a. Upper panel: input for endogenous TfR and overexpressed TfR/GFP. Lower panels: IP with anti-GFP antibodies and IB with anti-TfR antibodies at short and long exposure times (TIFF 1703 kb)
