# Supplementary figures and images for: MT5-MMP is a new pro-amyloidogenic proteinase that promotes amyloid pathology and cognitive decline in a transgenic mouse model of Alzheimer’s disease
Source: Cell Mol Life Sci. 2015 Jul 23;73(1):217–36. doi: 10.1007/s00018-015-1992-1 (PMC4700096; doi:10.1007/s00018-015-1992-1)

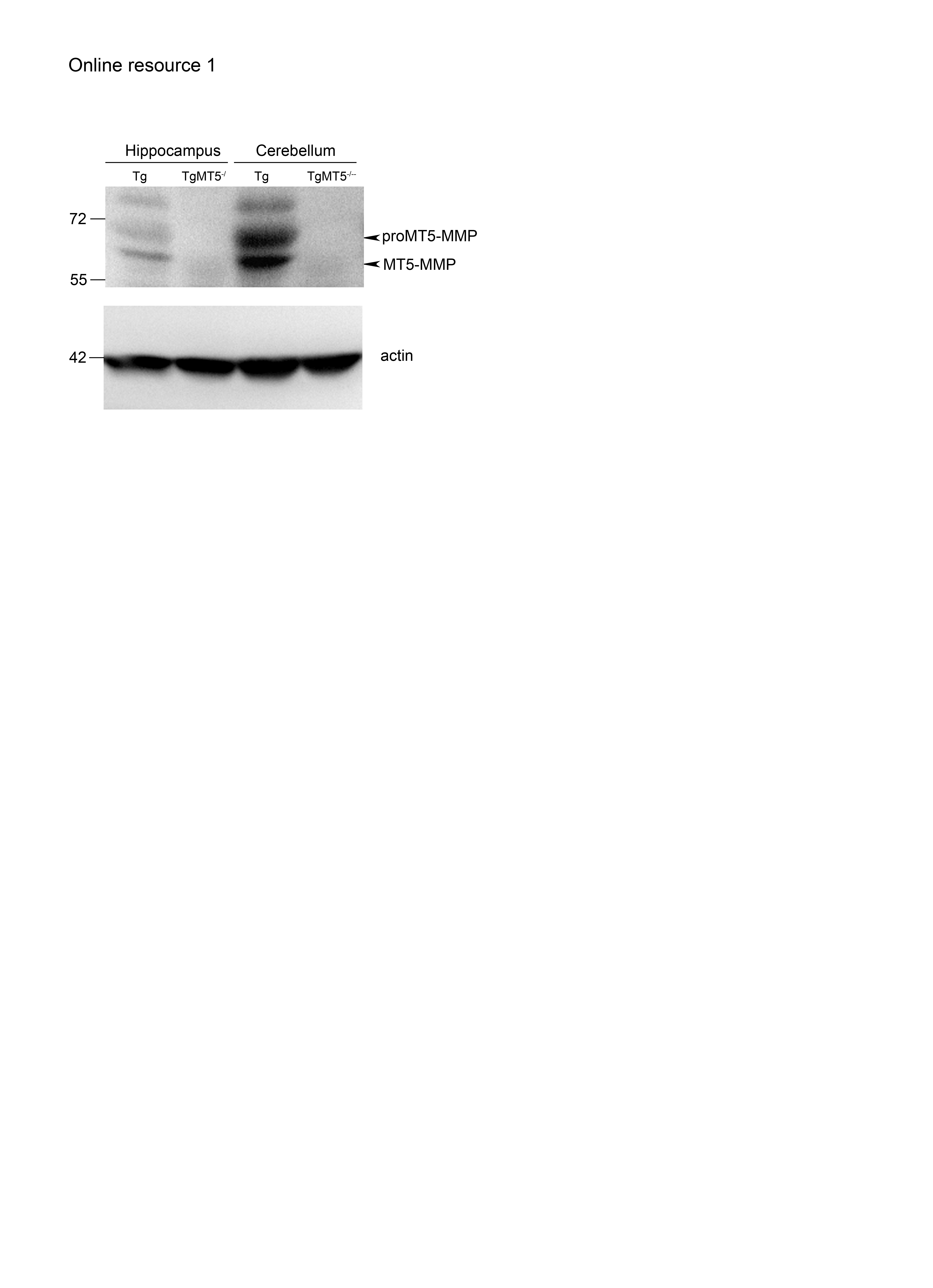

Supplement: Supplementary file 1 — Online resource 1. Efficient knock out of MT5-MMP in TgMT5−/− brains. Western blot using our own developed specific rabbit anti-mouse polyclonal MT5-MMP antibody in homogenates of hippocampus and cerebellum from P15 Tg and TgMT5−/− mice showing efficient knock out of MT5-MMP (TIFF 628 kb) [file 18_2015_1992_MOESM1_ESM.tif]

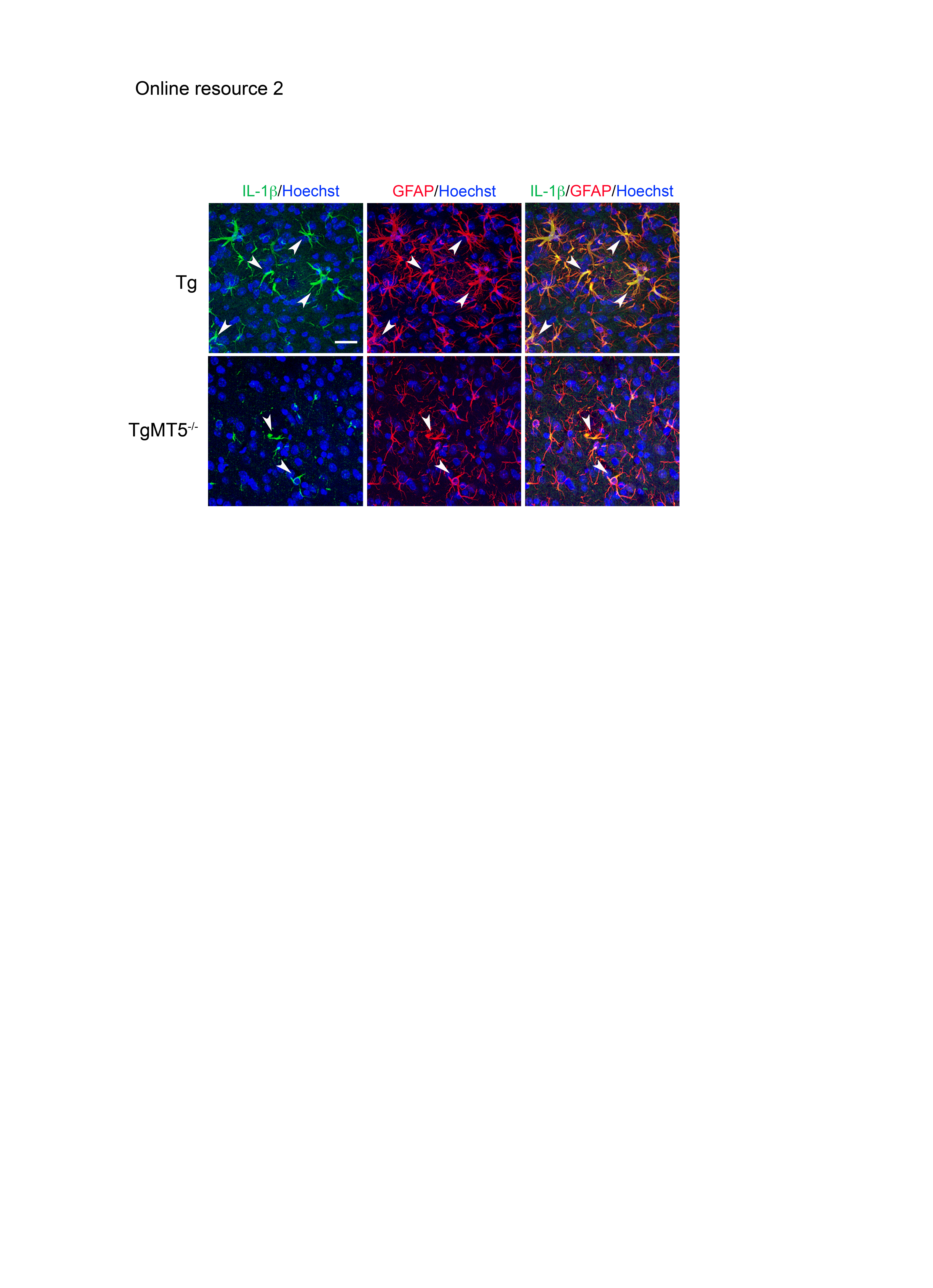

Supplement: Supplementary file 2 — Online resource 2. Astrocytic expression of IL-1β and strong reduction of IL-1β immunostaining in TgMT5−/− astrocytes. Confocal microphotographs representative of 7-8 mice per group showing double immunostained cortical sections with co-localization (yellow-arrowheads) of IL-1β (green) and GFAP (red) in the neocortex of Tg and TgMT5−/− mice. Note also the strong reduction in IL-1β immunostaining in TgMT5−/− brains compared to Tg. Scale bar : 20 µm (TIFF 8418 kb) [file 18_2015_1992_MOESM2_ESM.tif]

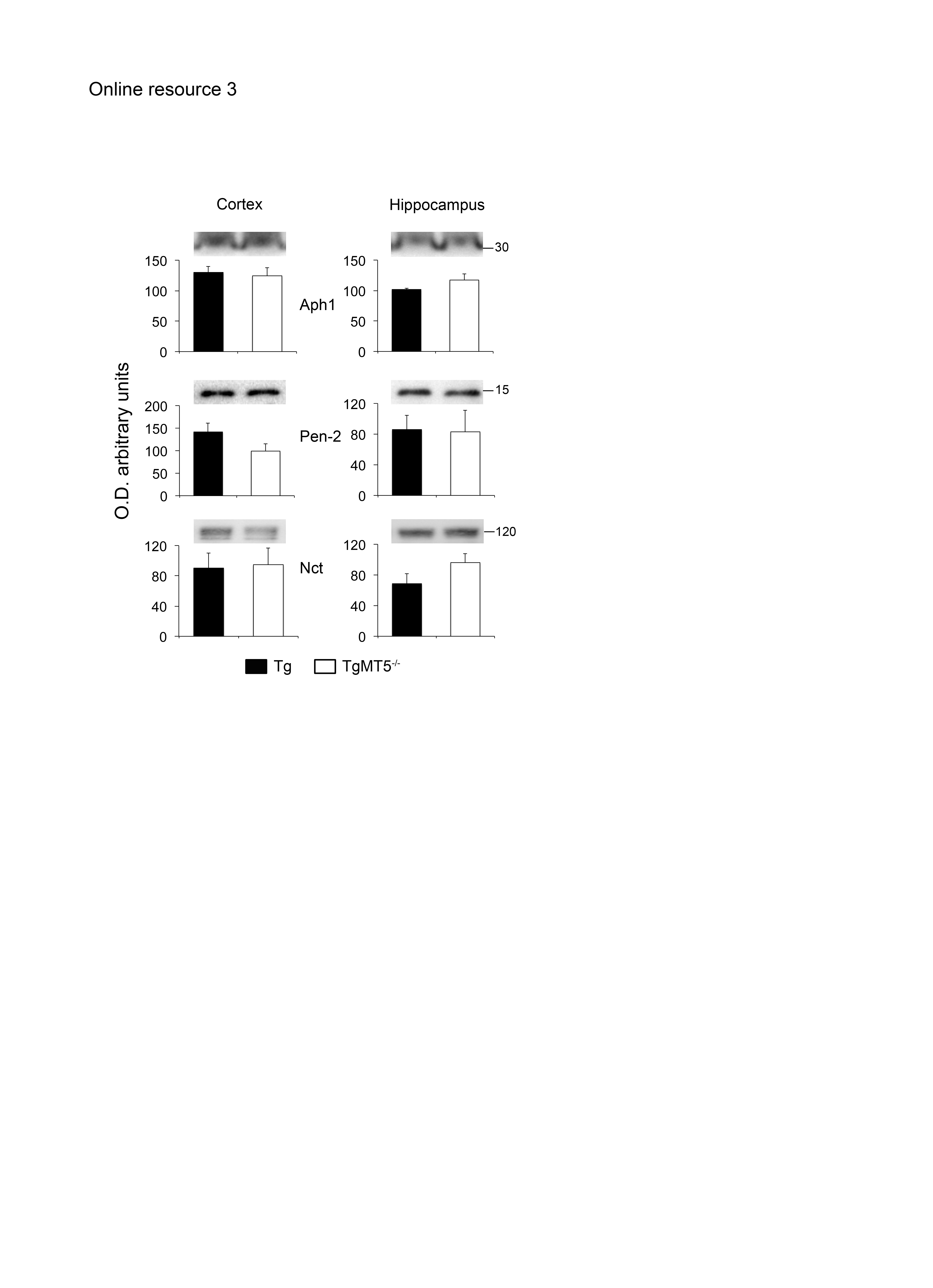

Supplement: Supplementary file 3 — Online resource 3. Expression of γ-secretase members Aph1, Pen-2 and nicastrin in the brains of Tg and TgMT5−/− mice. Representative western blots of Aph1, Pen-2 and nicastrin (Nct) in cortical and hippocampal homogenates, showing no differences between TgMT5−/− and Tg brains. Values represent the mean ± SEM of tubulin normalized optical densities (O.D.) from 7-8 brains per group (TIFF 380 kb) [file 18_2015_1992_MOESM3_ESM.tif]

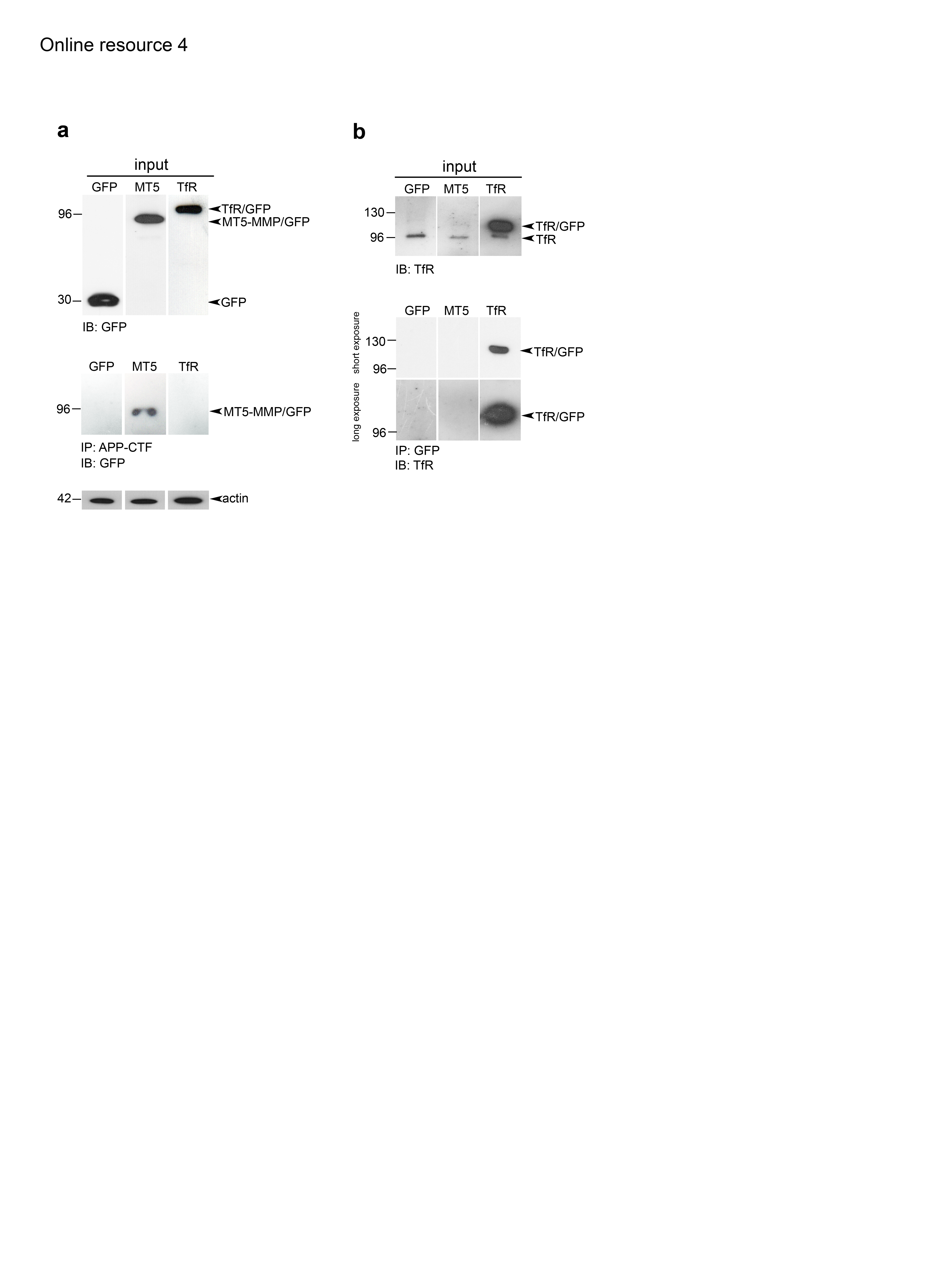

Supplement: Supplementary file 4 — Online resource 4. MT5-MMP/GFP and APP do not interact with endogenous or overexpressed transferrin receptor. a. Western blots representative of 4 independent cultures showing that immunoprecipitated APP interacts with MT5-MMP/GFP, but not with the overexpressed transferrin receptor (TfR) in lysates of cultured HEKswe cells 48 h after transfection with GFP control, MT5-MMP/GFP (MT5) or TfR/GFP (TfR) plasmids. Upper panel: input for overexpressed plasmids immunoblotted (IB) with anti-GFP antibodies. Lower panel: immunoprecipitation (IP) of endogenous APP with APP-CTF antibodies and IB with anti-GFP antibodies. Actin loading controls are representative of all inputs in a and b. b. Western blots repesentative of 4 independent cultures showing that MT5-MMP/GFP does not interact with TfR in the same experimental conditions described in a. Upper panel: input for endogenous TfR and overexpressed TfR/GFP. Lower panels: IP with anti-GFP antibodies and IB with anti-TfR antibodies at short and long exposure times (TIFF 1703 kb) [file 18_2015_1992_MOESM4_ESM.tif]
